# Identifying and analyzing the key genes shared by papillary thyroid carcinoma and Hashimoto’s thyroiditis using bioinformatics methods

**DOI:** 10.3389/fendo.2023.1140094

**Published:** 2023-05-31

**Authors:** Ting-ting Liu, De-tao Yin, Nan Wang, Na Li, Gang Dong, Meng-fan Peng

**Affiliations:** ^1^ Department of Ultrasound, The First Affiliated Hospital of Zhengzhou University, Zhengzhou, China; ^2^ Department of Thyroid Surgery, The First Affiliated Hospital of Zhengzhou University, Zhengzhou, China; ^3^ Engineering Research Center of Multidisciplinary Diagnosis and Treatment of Thyroid Cancer of Henan Province, Zhengzhou, China; ^4^ Key Medicine Laboratory of Thyroid Cancer of Henan Province, Zhengzhou, China; ^5^ Department of Breast Surgery, The First Affiliated Hospital of Zhengzhou University, Zhengzhou, China

**Keywords:** Hashimoto’s thyroiditis, papillary thyroid carcinoma, key genes, SERPINA1, LPAR5, ABR

## Abstract

**Background:**

Hashimoto’s thyroiditis (HT) is a chronic autoimmune disease that poses a risk factor for papillary thyroid carcinoma (PTC). The present study aimed to identify the key genes shared by HT and PTC for advancing the current understanding of their shared pathogenesis and molecular mechanisms.

**Methods:**

HT- and PTC-related datasets (GSE138198 and GSE33630, respectively) were retrieved from the Gene Expression Omnibus (GEO) database. Genes significantly related to the PTC phenotype were identified using weighted gene co-expression network analysis (WGCNA). Differentially expressed genes (DEGs) were identified between PTC and healthy samples from GSE33630, and between HT and normal samples from GSE138198. Subsequently, functional enrichment analysis was performed using Gene Ontology (GO) and Kyoto Encyclopedia of Genes and Genomes (KEGG). Transcription factors and miRNAs regulating the common genes in PTC and HT were forecasted using the Harmonizome and miRWalk databases, respectively, and drugs targeting these genes were investigated using the Drug-Gene Interaction Database (DGIdb). The key genes in both GSE138198 and GSE33630 were further identified *via* Receiver Operating Characteristic (ROC) analysis. The expression of key genes was verified in external validation set and clinical samples using quantitative real-time polymerase chain reaction (qRT-PCR) and immunohistochemistry (IHC).

**Results:**

In total, 690 and 1945 DEGs were associated with PTC and HT, respectively; of these, 56 were shared and exhibited excellent predictive accuracy in the GSE138198 and GSE33630 cohorts. Notably, four genes, Alcohol Dehydrogenase 1B (*ADH1B*), Active BCR-related (*ABR*), alpha-1 antitrypsin (*SERPINA1*), and lysophosphatidic acid receptor 5 (*LPAR5*) were recognized as key genes shared by HT and PTC. Subsequently, *EGR1* was identified as a common transcription factor regulating *ABR, SERPINA1*, and *LPAR5* expression. These findings were confirmed using qRT-PCR and immunohistochemical analysis.

**Conclusion:**

Four (*ADH1B, ABR, SERPINA1*, and *LPAR5*) out of 56 common genes exhibited diagnostic potential in HT and PTC. Notably, this study, for the first time, defined the close relationship between ABR and HT/PTC progression. Overall, this study provides a basis for understanding the shared pathogenesis and underlying molecular mechanisms of HT and PTC, which might help improve patient diagnosis and prognosis.

## 1 Introduction

Hashimoto’s thyroiditis (HT), also known as chronic lymphocytic thyroiditis, is an autoimmune disorder characterized by attack and destruction of the thyroid gland by the immune system (1). Although the etiology of HT is known to be complex, including genetic, environmental, and epigenetic factors, the precise associated mechanisms remain unclear (2). Moreover, HT is associated with an increased risk of various malignant tumors, particularly papillary thyroid carcinoma (PTC) (3). Indeed, multiple studies have proposed a relationship between HT and possible malignant transformation involving immunological and endocrinological pathogenic links. Thus, chronic inflammation might function as an important inducer of cellular transformation and tumor progression in the thyroid (4).

Thyroid carcinoma is the most prevalent endocrine malignant neoplasm worldwide, which includes papillary thyroid carcinoma (PTC), medullary thyroid carcinoma (MTC), anaplastic thyroid carcinoma (ATC), and follicular thyroid carcinoma (FTC) (5). The incidence of PTC has increased in recent years, making it the most common type of thyroid cancer, accounting for approximately 70% of all cases (6). Several well-known risk factors contribute to the development and progression of PTC, including exposure to ionizing radiation (7). However, other risk factors, including sex, obesity, diabetes, smoking, alcohol consumption, and genetic factors, have also been described (8). Meanwhile, compared to patients with PTC but without HT, patients with both PTC and HT exhibited slower tumor growth, less lymphatic spread, and less distant metastasis, possibly because of lymphatic infiltration into the tumor site (9). Therefore, researchers have become increasingly interested in characterizing the factors driving, or the mechanisms underlying, the transformation of chronic inflammation into thyroid malignancy (10, 11).

Since 1955, when the association between HT and PTC was first described (12), numerous epidemiological studies have confirmed the high degree of coexistence between HT and PTC, ranging from 20–85% (13–16). Indeed, the incidence of PTC in patients with HT is several times higher than that in patients without HT (13–16). Further, despite the clear relationship defined between HT and PTC, no such association has been reported between HT and other thyroid cancers, including MTC, ATC, and FTC (17). Paradoxically, in addition to its role in PTC development, HT has also been shown to protect against PTC progression. More specifically, the prognosis and clinicopathological features of patients with both PTC and HT are better than those of patients without HT. Approximately 18.9–23.2% of patients with PTC are reported to have HT, and show a better prognosis than that of patients without HT (14, 18). However, the association between HT and PTC remains controversial, with some arguing that patients with HT undergo more frequent screening, which confounds this association (19). Therefore, identifying the key genes shared by HT and PTC can provide important insights regarding their shared pathogenesis and the molecular mechanisms underlying the development of HT and PTC.

Bioinformatics has been widely employed to analyze diagnostic and therapeutic targets in various diseases (19, 20). In this study, we aimed to screen the key genes shared by HT and PTC, based on a series of bioinformatics analyses using the GSE33630 and GSE138198 datasets (21, 22), along with analyses of the regulatory mechanisms and potential drugs targeting these genes. Subsequently, quantitative real-time polymerase chain reaction (qRT-PCR) and immunohistochemical analysis of clinical PTC samples in HT was performed to confirm the expression trends of the key genes. We believe that this study provides theoretical support for continued investigation into the shared pathogenesis and molecular mechanisms of PTC and HT, and thereby help improves the diagnosis and prognosis of patients with these diseases.

## 2 Materials and methods

### 2.1 Dataset collection

In this study, the Gene Expression Omnibus (GEO) database (https://www.ncbi.nlm.nih.gov/geo/) was queried using the keywords ‘Hashimoto’s thyroiditis’ and ‘papillary thyroid carcinoma’ to retrieve two datasets for HT and PTC, respectively. In our analysis, we used the transcriptome sequencing data of 45 normal thyroid tissues and 49 PTC tissue samples from the GSE33630 dataset. Further, the transcriptome sequence data of three normal thyroid tissues, 13 HT samples, and eight PTC samples with HT disease background from the GSE138198 dataset were included in our study. RNA sequencing data in thyroid tumors related to The Cancer Genome Atlas (TCGA) cohorts was utilized as an external validation set for the key gene expression (23), including 513 tumor samples and 59 control samples.

### 2.2 Analysis for the differentially expressed genes (DEGs) in PTC and HT

Based on the data of 45 normal thyroid tissue and 49 PTC tissue samples from GSE33630 dataset, the R package ‘WGCNA’ (24) was used to generate a signed co-expression network for filtering PTC-related genes that were significantly relevant to the PTC phenotype using weighted gene co-expression network analysis (WGCNA). After assessing the presence of outlier samples using unsupervised clustering, we selected an optimal soft-thresholding parameter of 11 to ensure a scale-free network, where scale free R^2^ = 0.85 and the mean connectivity was close to 0. We then transformed the adjacency matrix into the Topological Overlap Measure (TOM), and conducted hierarchical clustering. Genes with similar expression profiles were classified into the same gene modules using the DynamicTreeCut algorithm with a minimum size of 200. Subsequently, the correlation of modules with traits was calculated and displayed as a heatmap, and the module with the strongest correlation was selected as the key module for further analysis.

Based on |log_2_FoldChange (FC)| > 1 and False discovery rate (FDR) < 0.05 (where *p*-values was adjusted with a Benjamini-Hochberg (BH) adjustment for multiple testing through the method of ‘fdr’ in the ‘p.adjust’ function) (25), we authenticated the differentially expressed PTC-related genes in GSE33630 dataset using the R package ‘limma’ package (26). Differentially expressed genes (DEGs) between 13 HT and 3 normal samples in the GSE138198 dataset were also mined using Wilcoxon test with |log_2_FC| > 1 and *p*-value < 0.05 as the filtering criteria.

### 2.3 Functional annotation analysis

The R package ‘clusterProfiler’ (27) was first used to explore the Gene Ontology (GO) and Kyoto Encyclopedia of Genes and Genomes (KEGG) enrichment terms relevant to the DEGs; GO terms were categorized into cellular components (CC), molecular functions (MF), and biological processes (BP). The significance criterion was adjusted to a *p*-value < 0.05.

### 2.4 Recognition of common key genes in HT and PTC

The differentially expressed PTC-related genes and DEGs in HT were intersected and presented in a Venn diagram, where the overlapped genes were considered as common genes and imported into Cytoscape to map the co-expression network with the biological terms using the ClueGO plug-in. The common genes were imported into Cytoscape, and the ClueGO plug-in was used to map the co-expression network between the biological terms and these genes. Furthermore, according to the gene expression patterns, the common genes were included in subsequent Receiver Operating Characteristic (ROC) analyses for identifying their predictive accuracy in GSE33630 (PTC vs Normal) and GSE138198 (HT vs Normal) datasets using the ‘pROC’ package, where the best threshold value was selected according to the Youden index. In addition, genes with area under the curve (AUC) values ≥ 0.95 in the GSE33630 and GSE138198 datasets were identified as common key genes shared by HT and PTC.

### 2.5 Establishment of the miRNA/TF-HT and PTC related genes-drug network

Key transcription factor (TF)-regulating genes were predicted using the Harmonizome database (http://amp.pharm.mssm.edu/Harmonizome) (CHEA Transcription Factor Targets dataset). The miRNAs targeting the common genes as well as key genes were predicted using the miRWalk database (http://mirwalk.umm.uni-heidelberg.de/) (screening condition: score > 0.9). Drugs targeting the common genes and key genes were predicted using the DGIdb (http://www.dgidb.org) database. The final miRNA/TF-key gene-drug network was mapped using Cytoscape software (28).

### 2.6 RNA acquisition and real-time quantitative PCR (qRT-PCR)

This study was approved by the Ethics Committee of the First Affiliated Hospital of Zhengzhou University and was conducted according to the principles of the Declaration of Helsinki. Written informed consent was obtained from each patient before sample collection. The tumor tissues and non-tumor adjacent tissues (NAT) collected from nine patients with PTC in HT to assess the expression of key genes. All patients underwent surgery at the First Affiliated Hospital of Zhengzhou University, Henan, China, between July 2021 and March 2022, without receiving any anticancer treatment before surgery. All patients were between 21 and 60 years old and consented to the postoperative follow-up plan (**Supplementary Table 1**). Tissue specimens were collected within 30 minutes of surgery and immediately frozen in liquid nitrogen. Postoperative monitoring and treatment were continued according to the relevant consensus guidelines. The degree of tumor differentiation was graded according to WHO grading system(29). Total RNA from nine NAT and nine PTC samples was isolated using TRIzol reagent, following the manufacturer’s instructions (Ambion, Austin, Texas, USA). Total RNA was reverse-transcribed into cDNA using a SweScript First Strand cDNA Synthesis Kit (Service bio, Wuhan, China), according to the manufacturer’s protocol. QPCR was subsequently carried out using the 2×Universal Blue SYBR Green qPCR Master Mix (Service bio, Wuhan, China), according to the manufacturer’s instructions. The primer sequences used for PCR are listed in **Table 1**. The relative expression level was compared to that of the internal reference gene (*GAPDH*) and calculated using the 2^−ΔΔCq^ method (30).

**Table 1:** The primer sequences used for RT-qPCR.

Primer	Sequence
ABR For	GCCGTCTTCGATGCCAATAAC
ABR Rev	TGGGTAGAGTCGGTCCGTGAG
ADH1B For	CATCAACCCTCAAGACTACAAGAA
ADH1B Rev	GCGTCCAGTCAGTAGCAGCATAG
LPAR5 For	TGCTGTGCTTCGTGCCCTAC
LPAR5 Rev	GCGGACCTTTCGGATTGC
SERPINA1 For	CGTGAAGGTGCCTATGATGAAG
SERPINA1 Rev	CCAGTAATGGACAGTTTGGGTAA
GAPDH For	CCCATCACCATCTTCCAGG
GAPDH Rev	CATCACGCCACAGTTTCCC

### 2.7 Immunohistochemistry (IHC)

We performed immunohistochemical analysis on the tumor tissues and NAT collected from eight patients with PTC in HT. The tissues were collected and fixed in 4% paraformaldehyde, embedded in paraffin, and sectioned into 5 µm thick slices. The sections were placed in EDTA (pH 9.0) for antigen repair, washed with phosphate-buffered saline (PBS, pH 7.4), treated with 3% H_2_O_2_, blocked with bovine serum albumin (BSA), and incubated overnight at 4 °C with antibodies specific to ABR (1:200; ab224129, Affinity, Changzhou, Jiangsu, China), ADH1B (1:250; bs-10591R, Bioss, Beijing, China), LPAR5 (1:200; bs-15366R, Bioss, Beijing, China), and SERPINA1 (1:200; bs-0096R, Bioss, Beijing, China). Subsequently, the samples were treated with a secondary goat anti-rabbit IgG antibody (1:200; DAKO) for 50 minutes at 37 °C, and the positive sites were labeled with DAB (diaminobenzidine) developing solution (G1211, Service bio, Wuhan, China). Finally, hematoxylin staining (G1004, Service bio, Wuhan, China) was performed to visualize the nuclei. Images (200× magnification) were captured with a microscope (OLYMPUS), and three different visual fields were analyzed. The calculation of the immune response score (IRS) is typically done manually by visual assessment by the operator or technician. IRS is calculated by multiplying the proportion score (ranging from 0 to 4) by the staining intensity score (ranging from 0 to 3) for each cell type evaluated according to the method proposed by Rem-mele and Stegner(31). The immunoreactivity score (IRS) was determined as IRS = SI × PP (staining intensity× percentage of positive cells) and was divided into four grades as follows: 0, non-staining means no positive staining (0–5%); 1, Light yellow means weak positive; 2, Brownish yellow means medium positive; 3, tan means strong positive. The cell ratio was then graded on a 0–4 scale (positive cell ratio = positive cell count/total cell count) as follows: 0–5%, grade 0; 6–25%, grade 1, 26–50%, grade 2; 51–75%, grade 3; and 75–100%, grade 4. The resulting scores for each cell type are then summed up to obtain the total IRS score, which can range from 0 to 12. When IRS score>3 is an immune response (+).

### 2.8 Statistical analysis

All analyses were conducted using R programming language. Data from different groups were compared using the Wilcoxon test or Student’s t-test. Unless otherwise specified, a *p*-value < 0.05 was considered statistically significant.

## 3 Results

### 3.1 Differentially expressed PTC-related genes

**Figure 1:**
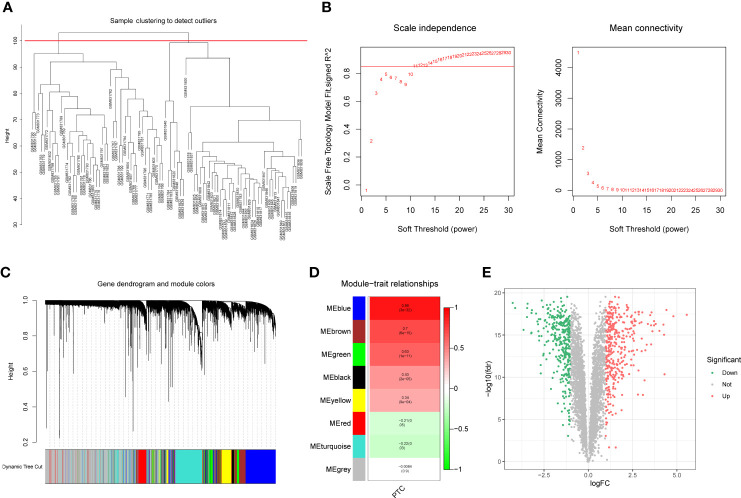
**(A)** Sample clustering and detection of outliers. **(B)** The scale-free fit index (scale-free R^2^, y-axis) as a function of the soft-threshold power (x-axis) and the mean connectivity of the network. **(C)** Gene expression dendrogram. The color annotations provide a simple visual comparison of module assignments (branch cuttings) based on the dynamic tree-cutting method. **(D)** Trait-module correlation presented in a Module-Trait Relationships chart; the blue module has the highest correlation and represents the key module. **(E)** A total of 690 genes were differentially expressed; 367 = (red) were upregulated, and 323 (blue) were downregulated.

To identify the differentially expressed PTC-related genes, WGCNA was performed using the GSE33630 dataset. The sample cluster analysis did not exclude outlier samples (**Figure 1A**). To ensure that the interactions between the genes maximally conformed to a scale-free distribution, the optimal soft threshold (R^2^ = 0.85) was selected as 11 (**Figure 1B**). Next, eight modules were developed based on a gene clustering tree and a dynamic tree-cutting algorithm (with a minimum of 200 genes per gene module) (**Figures 1C, D**). Following correlation analysis between the modules and sample traits (normal or PTC), the blue module with the highest correlation was selected as the key module (**Figure 1D**). Ultimately, 4235 genes in the key module were considered PTC-related genes (**Supplementary Table 2**). Based on these, differential analysis between the PTC and normal groups was performed. In total, 690 divergent genes, including 367 with increased expression and 323 with decreased expression in PTC samples, were characterized (**Figure 1E**; **Supplementary Table 3**).

**Figure 2:**
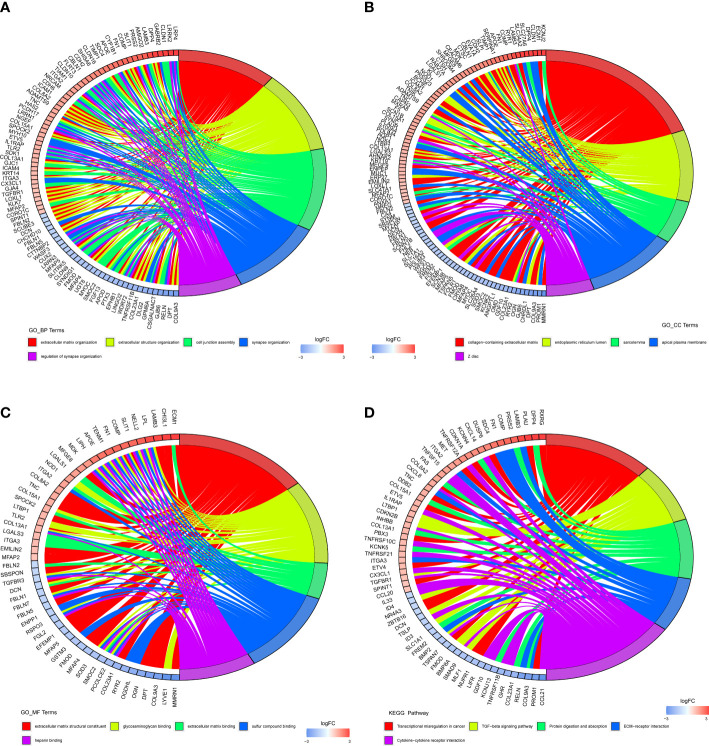
Functional enrichment analysis of differentially expressed genes (DEGs) in papillary thyroid carcinoma (PTC). **(A)** Chord plot of the top five Gene Ontology (GO) biological processes (BP) enriched pathways. **(B)** Chord plot of the top five GO cellular components (CC) enriched pathways. **(C)** Chord plot of the top five GO molecular functions (MF) enriched pathways. **(D)** Chord plot of the top five Kyoto Encyclopedia of Genes and Genomes (KEGG) enriched pathways.

Functional enrichment analysis was performed to further investigate the functions of the 690 differentially expressed PTC-related genes. As shown in **Supplementary Table 4**, 286 GO items (229 BP items, 43 CC items, and 14 MF items) and nine KEGG pathways were identified. The top five items in each category were displayed as chordal graphs (**Figure 2**). These genes have been implicated in synapse-related biological processes, cell-substrate adhesion, regulation of cell adhesion, hormone metabolic processes, thyroid hormone generation, thyroid hormone metabolic processes, and immune-related biological processes. Moreover, these genes were found to be associated with transcriptional dysregulation in cancer, as well as the TGF-β signaling pathway, ECM-receptor interaction, cytokine-cytokine receptor interaction, complement and coagulation cascades, and p53 signaling pathway.

### 3.2 DEGs in HT

**Figure 3:**
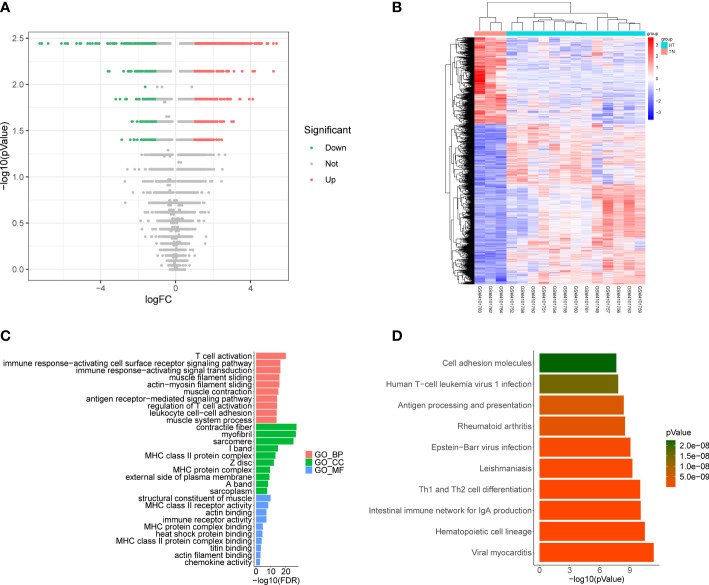
**(A)** Volcano plot of differentially expressed genes (DEGs) between Hashimoto’s thyroiditis (HT) samples and normal samples in the GSE138198 dataset through Wilcoxon test; red points represent upregulated DEGs, and green points represent downregulated DEGs. **(B)** Heatmap of DEGs between HT samples and normal samples in the GSE138198 dataset; the red squares represent the upregulated DEGs and the blue squares represent the downregulated DEGs. **(C)** Bar plot of the enriched GO terms of HT DEGs. **(D)** Bar plot of the enriched Kyoto Encyclopedia of Genes and Genomes (KEGG) pathways of HT DEGs.

Differential expression analysis conducted using the GSE138198 dataset to determine DEGs between HT and normal samples, ultimately revealed 1945 DEGs, including 1264 that were upregulated and 681 that were downregulated in HT samples (**Figures 3A, B**, **Supplementary Table 5**). Functional enrichment analysis further revealed 834 GO terms (748 BP, 59 CC, and 27 MF), and 52 KEGG pathways (**Supplementary Table 6**). The top ten items in each classification are displayed in chordal graphs in **Figures 3C, D**, these genes were primarily involved in biological processes related to the immune system, oxidative stress, and cell adhesion. Additionally, they were implicated in immune-related pathways, cell adhesion molecules, autoimmune thyroid disease, phagosome, Fc gamma R-mediated phagocytosis, the PPAR signaling pathway, and the FoxO signaling pathway.

### 3.3 Analysis for common genes in PTC and HT

**Figure 4:**
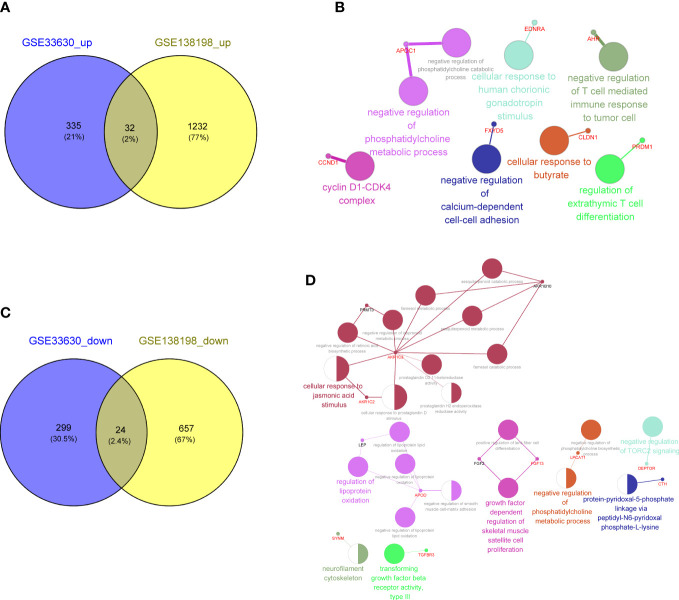
**(A)** Venn diagram showing 32 common upregulated differentially expressed genes (DEGs) shared by papillary thyroid carcinoma (PTC) and Hashimoto’s thyroiditis (HT). **(B)** Gene Ontology (GO) and Kyoto Encyclopedia of Genes and Genomes (KEGG) enrichment terms of the 32 common upregulated DEGs shared by PTC and HT visualized using the ClueGO plugin in Cytoscape software. **(C)** Venn diagram showing the 24 common downregulated DGEs shared by PTC and HT. **(D)** Function enrichment results of 24 common downregulated DGEs shared by PTC and HT.

To explore the key genes shared between PTC and HT, we first compared the DEGs related to PTC-and HT, yielding 32 upregulated and 24 downregulated genes shared among the PTC and HT samples (**Figures 4A, C**; **Supplementary Table 7**). The functions and pathways of these 56 differential genes were further investigated by constructing a co-expression network using the ClueGO plug-in in Cytoscape software, which further confirmed that the upregulated common genes were associated with negative regulation of T cell mediated immune response to tumor cell (**Figure 4B**; **Supplementary Table 8**). Simultaneously, the downregulated common genes were related to negative regulation of phosphatidylcholine biosynthesis (**Figure 4D**; **Supplementary Table 9**).

The prognostic value of these genes was also evaluated in subsequent ROC analysis using the GSE33630 and GSE138198 datasets. It was found that the AUC value of the 56 common genes was greater than 0.8, revealing excellent predictive accuracy in PTC and HT (**Supplementary Table 10**).

**Figure 5:**
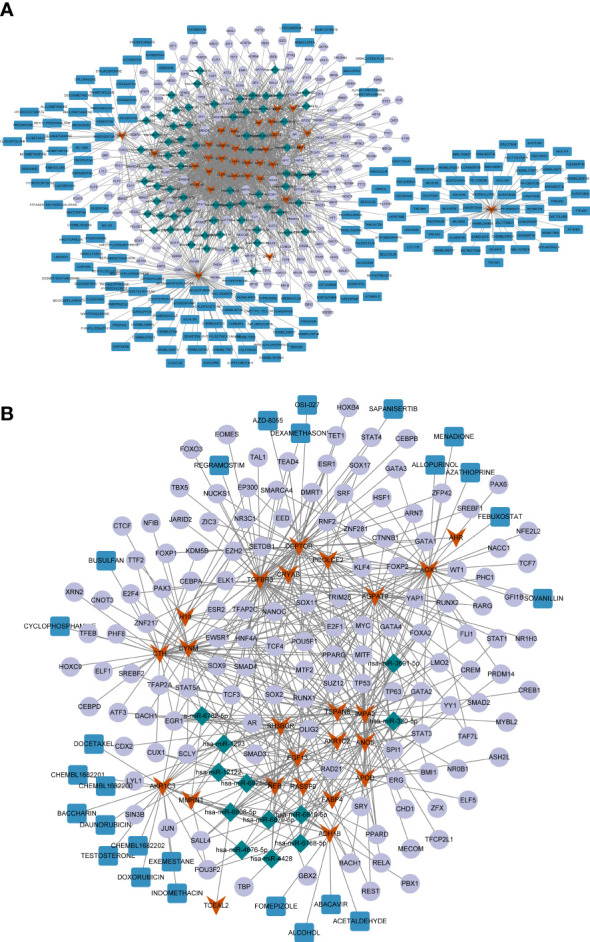
**(A)** The miRNA/TF-key gene-drug network constructed based on 32 common upregulated differentially expressed genes (DEGs) shared by papillary thyroid carcinoma (PTC) and Hashimoto’s thyroiditis (HT). **(B)** The miRNA/TF-key gene-drug network constructed based on 24 common downregulated DGEs shared by PTC and HT. The red arrows represent the common DEGs, the green diamonds represent miRNAs, the purple circles represent the transcription factors (TFs), and the red rectangles represent the compounds.

Considering the important regulatory roles of various miRNAs and TFs in PTC progression, we performed miRNA and TF prediction for these common genes to explore their upstream regulators. Using the miRWalk and Harmonizome databases, the miRNAs/TFs-genes networks targeting the upregulated and downregulated common genes were identified (**Supplementary Tables 11**, **12**), respectively. As shown in **Figure 5A**, the expression of many upregulated genes was regulated by TF *SOX2* and *hsa-miR-7110-5p*. Meanwhile, *HNF4A* was the common TF binding to various downregulated genes, such as *DEPTOR* and *TGFBR3* (**Figure 5B**), and *ADH1B* was connected with many other genes, such as *hsa-miR-6879-5p* and *hsa-miR-330-5p*.

Next, potential therapeutic agents or molecular compounds targeting the common genes were identified for PTC and HT treatment using the DGIdB. The results revealed plenty of compounds that could target upregulated *AURKB*, including the HER inhibitor LAPATINIB, which can target both *AURKB* and *CCND1* (**Figure 5A**). Additionally, the downregulated *AKR1C3* and *ADH1B* could be targeted by several compounds including DOXORUBICIN and ALCOHOL (**Figure 5B**).

### 3.4 Identification of key genes in PTC and HT

**Figure 6:**
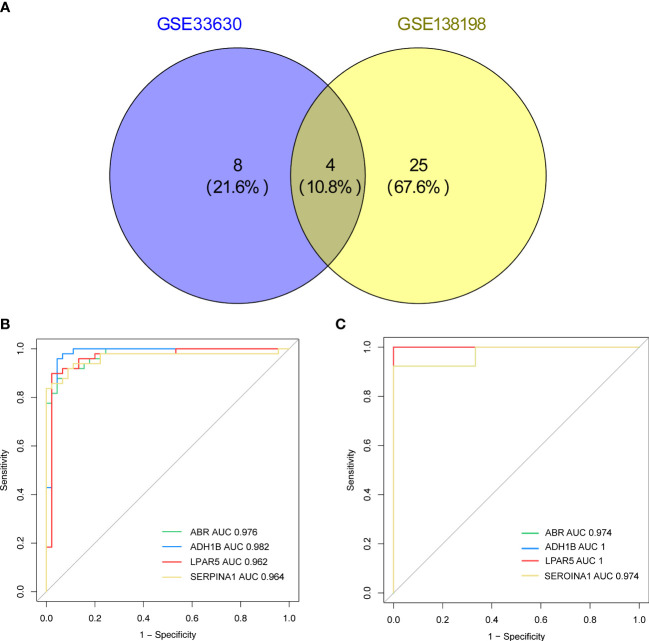
**(A)** Venn diagram showing the key genes of Hashimoto’s thyroiditis (HT) and papillary thyroid carcinoma (PTC) with area under the curve (AUC) values > 0.95 in the GSE33630 and GSE138198 datasets. **(B)** Receiver operating characteristic **(**ROC) curves of the four key genes (*ADH1B, ABR, SERPINA1, LPAR5*) in the HT and PTC in the GSE33630 dataset. **(C)** ROC curves of the four key genes (*ADH1B, ABR, SERPINA1*, and *LPAR5*) in HT and PTC in the GSE138198 dataset.

Based on the screening criterion of AUC values ≥ 0.95, four genes (*ADH1B, ABR, SERPINA1*, and *LPAR5*) in both the GSE33630 and GSE138198 datasets were identified as common key genes in HT and PTC (**Supplementary Table 10**; **Figures 6A–C**), and the Youden’s index for each key gene was calculated in **Figure S1**.

A miRNA/TF-key gene-drug network was generated for the four key genes (**Figure S2**). Notably, most miRNAs targeted ABR, including *miR-145-5p, miR-506-3p, miR-34a*, and *miR-449a*. Among the potential TFs, *EGR1* was the common target of *SERPINA1, ABR*, and *LPAR5*. *YY1* and *hsa-miR-297* were potential *ADH1B* targets, while *SERPINA1* and *ABR* were regulated by *MYC, AR*, and *ESR1*. Additionally, *SERPINA1* and *LPAR5* expression may be affected by *RUNX1* and *SMAD3*. With respect to the potential molecular compounds targeting the key genes, the results revealed four compounds that could target *ADH1B* (acetaldehyde, fomepizole, alcohol, and abacavir). The alpha-1 antitrypsin inhibitor, IGMESINE (C23H29N) may target *SERPINA1*. Furthermore, two compounds, arachidonoyl glycine and CHEMBL1630084, could target *LPAR5* (**Figure S2**). However, information for *ABR* was lacking in DGIdb.

### 3.5 Verification of key gene expression in clinical samples

**Figure 7:**
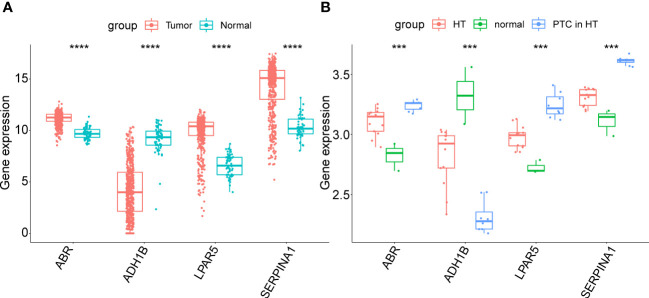
**(A)** Expression of key genes in PTC samples compared to normal samples in the TGCA-PTC dataset. **(B)** Expression of key genes in three normal thyroid tissue samples, 13 Hashimoto’s thyroiditis (HT) samples and eight papillary thyroid carcinoma (PTC) samples with HT disease background in the GSE138198 dataset. The asterisks represent the significance of the difference, the more asterisks, the more significant the difference.

Next, the expression patterns of the four key genes were assessed in external validated cohorts (including TCGA-PTC cohorts, GSE138198 dataset and clinical samples with PTC in HT). In the TCGA-PTC dataset three key genes, *ABR*, *LPAR5*, and *SERPINA1*, were found upregulated in PTC samples compared with those in normal samples. In contrast, *ADH1B* expression was downregulated in PTC samples compared to normal samples (**Figure 7A**). This trend was shown by the expression data from GSE138198 cohort (**Figure 7B**). Notably, it can be seen that changes in gene expression in PTC samples with HT background were more pronounced than that in HT samples, indicating the potential synergies between HT and PTC progression.

**Figure 8:**
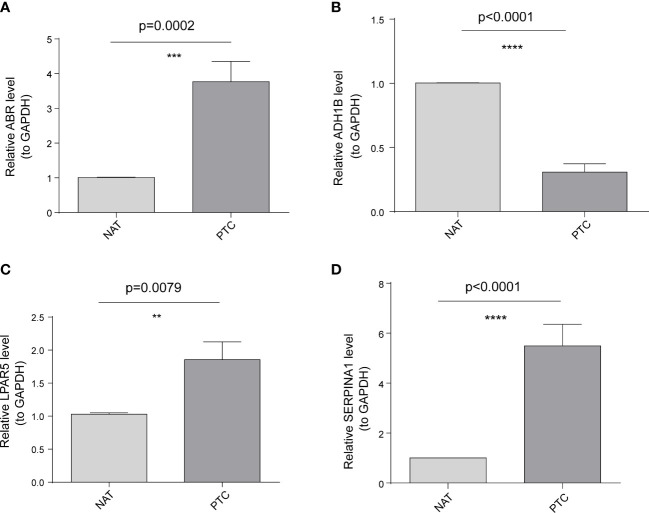
Results of the public database data analysis confirmed using qRT-PCR. In nine papillary thyroid carcinoma (PTC) samples with Hashimoto’s thyroiditis (HT) disease. **(A)** ABR was significantly upregulated in cancer tissues (PTC) compared to non-tumor adjacent tissue (NAT). **(B)** ADH1B was significantly downregulated in PTC compared to NAT. **(C)** LPAR5 was significantly upregulated in PTC compared to NAT. **(D)** SERPINA1 was significantly upregulated in PTC compared to NAT. The asterisks represent the significance of the difference, the more asterisks, the more significant the difference.

**Figure 9:**
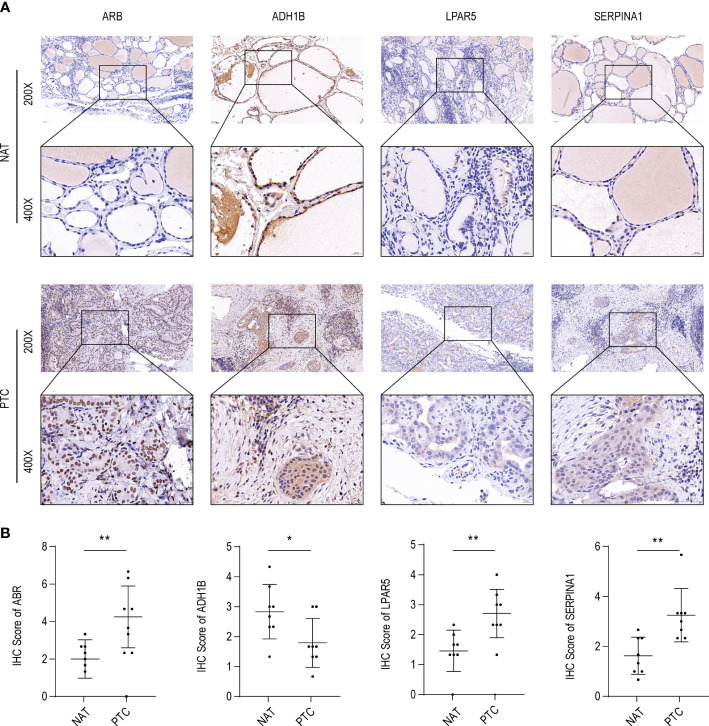
ABR, ADH1B, LPAR5, and SERPINA1 abundance in papillary thyroid carcinoma (PTC). **(A)** Immunohistochemical (IHC) analysis of ABR, ADH1B, LPAR5, and SERPINA1 abundance in cancer tissues (PTC) and non-tumor adjacent tissue (NAT) of eight PTC samples with Hashimoto’s thyroiditis (HT) disease. **(B)** IHC scores of ABR, ADH1B, LPAR5, and SERPINA1 in PTC and NAT. The asterisks represent the significance of the difference, the more asterisks, the more significant the difference.

To further validate these expression patterns, we performed RT-qPCR on tumor tissues and NAT collected from nine patients with PTC in HT. Consistent with the bioinformatics data analysis results, *ABR, LPAR5*, and *SERPINA1* were significantly upregulated in clinical PTC samples compared to NAT, whereas *ADH1B* was significantly downregulated (**Figure 8**). Additionally, we assessed the expression of the key genes in tumor tissues and NAT collected from eight patients with PTC in HT using IHC. In agreement with the bioinformatics data analysis results, the abundance of *ABR, LPAR5*, and *SERPINA1* was significantly upregulated in clinical PTC samples compared to NAT, whereas *ADH1B* was significantly downregulated (**Figure 9**).

## 4 Discussion

We assessed differences in gene expression between PTC, HT, and normal thyroid samples. First, we identified 690 divergent genes between PTC and normal thyroid samples. Functional enrichment analysis revealed that PTC occurrence may be associated with synapse-related biological and hormone metabolic processes, cell-substrate adhesion, cell adhesion regulation, p53 and TGF-β signaling, and immune-related biological processes. Inflammatory mediators contribute to tumor progression via myriad mechanisms (32). Subsequently, 1945 DEGs were detected between HT and normal samples in the GSE138198 dataset. These genes were implicated in immune-related and oxidative stress-related pathways, phagosomes, Fc gamma R-mediated phagocytosis, the PPAR and FoxO signaling pathways, and cell adhesion-related biological processes. Thus, the biological pathways associated with the DEGs of HT and PTC are closely related. Although HT etiology has not yet been fully elucidated, it is associated with the release of cytokines that can damage thyroid follicular cells following helper T lymphocyte activation (33, 34). Inflammatory cytokines, such as tumor necrosis factor-α, IL-6, and IL-1, are associated with COX-2 expression, which is related to various malignancies (34, 35). Moreover, cross-reactivity with lactoperoxidase, that induces chronic inflammation in HT, can promote PTC (36). Chronic inflammation in turn leads to matrix remodeling, resulting in changes in cell adhesion, DNA damage, genetic alterations (such as *p53* mutations), and protective antitumor immunosuppression (37–39). Therefore, we suggest that chronic inflammation represents a possible mechanism of PTC owing to its effect on the immune microenvironment and tumor suppressor gene mutation induction (32).

There were 56 common genes in PTC and HT, of which the 32 up-regulated genes were related to the negative regulation of T cell-mediated tumor cell immune response, while the 24 down-regulated genes were related to the negative regulation of phosphatidylcholine biosynthesis. HT is an autoimmune disease mainly mediated by T cells (40). Phosphatidylcholine synthesis plays an important role in HT (41), but these pathways have not been reported in PTC. Due to the strong correlation between PTC and HT, these pathways can provide new research directions for future PTC studies.

In addition to ROCAUC analysis of the 56 common genes, we further investigated the transcriptional regulation and drugs targeting these genes to gain a deeper understanding of the progression and potential therapeutic mechanisms of PTC. Increased expression of SOX2 promotes the progression of PTC (42, 43). MiR-330-5p can promote the progression of PTC (44) by upregulating ETV4. These genes were affected by competing endogenous RNA (ceRNA) regulatory mechanisms, which may provide new therapeutic targets for the treatment of PTC. Furthermore, miR-6879-5p, as a key exocrine marker, is associated with the progression of PTC (44). Here, the target gene of miR-330-5p and miR-6879-5p was ADH1B, both of which have been reported in PTC, but their interaction mechanism has not been studied, and further exploration is warranted. Previous bioinformatics analysis (45) showed that HNF4A has a significant effect on the progression of PTC, but the mechanism of its interaction with the target gene SERPINA1 has not been investigated. Our results indicate that HNF4A is a common TF-binding site for many downregulated genes. However, functional analysis of these genes awaits further study. Further investigation of these regulatory mechanisms could elucidate the underlying molecular mechanisms of PTC progression. In our drug-prediction analysis, we focused on two genes, CCND1 and AKR1C3, which have been implicated in PTC pathogenesis. LAPATINIB has been studied in the treatment of PTC drug resistance as a HER inhibitor (46). In addition, LAPATINIB has been shown to reduce the expression of CCND1 in breast cancer (47). Here, the expression of CCND1 increased, suggesting that LAPATINIB could be a potential therapeutic agent for PTC by targeting CCND1. Moreover, we analyzed the interaction between AKR1C3 and DOXORUBICIN, a commonly used chemotherapy drug for PTC, and found that targeting AKR1C3 might inhibit DOXORUBICIN drug resistance in PTC cells and enhance the efficacy of DOXORUBICIN treatment in PTC (48, 49).

ROC analysis of the 56 genes from the GSE33630 and GSE138198 datasets revealed four key genes shared by HT and PTC with AUC values of > 0.95 (i.e., *ADH1B, ABR, SERPINA1*, and *LPAR5*). *ADH1B* is related to many phenotypic traits, including alcohol metabolism, liver function, and cancer (50, 51). Moreover, single nucleotide polymorphisms (SNPs) in *ADH1B* have been correlated with esophageal square cell carcinoma (ESCC), colorectal cancer (CRC), and overall cancer. However, *ADH1B* SNPs are not associated with PTC or HT (51–55). Furthermore, *ADH1B* is primarily a metabolism-related gene that is involved in the physiological processes of ethanol, uric acid, fat, and glucose metabolism as well as in the regulation of peripheral blood lymphocyte proliferation and nerve axons protection(56–58). ADH1B serves as an autotarget antigen for Graves’ disease, an autoimmune inflammatory disease (59). Therefore, we posit that *ADH1B* may represent the key gene responsible for HT and PTC interaction.

*ABR*, located on chromosome 17p, is closely associated with chronic and acute myeloid and acute lymphoblastic leukemias (60, 61). ABR protein expression levels are the highest in the central nervous system and may interact simultaneously or sequentially with members of the Rho family to regulate and coordinate cellular signaling (62, 63). To the best of our knowledge, this study is the first, to report a relationship between *ABR* and HT or PTC. We hypothesize that *ABR* may influence HT or PTC development *via* myelination regulation.

SERPINA1, a protein associated with inflammatory disease, organismal injury, and abnormal thyroid function, is significantly lower in hyperthyroidism samples than in normal thyroid samples (64, 65). As a hub gene for PTC-DEGs, *SERPINA1* is potential diagnostic gene for PTC and is related to the extracellular matrix pathway (66–68). Our findings further suggest that SERPINA1 serves as an important cross-linking gene involved in the pathogenesis of HT and PTC.

Lysophosphatidic acid receptor 5 (LPAR5) overexpression is involved in mediating thyroid cancer progression; however, the mechanism underlying this process requires further elucidation (69, 70). Additionally, LPAR5 is reportedly associated with lysophosphatidic acid-induced pro-inflammatory signaling cascades in microglia (71–73). Therefore, LPAR5 may be involved in a common pathway between HT and PTC, which represents a promising area of future investigation.

Furthermore, interaction networks containing 88 nodes and 100 edges were constructed to identify potential regulatory mechanisms targeting the four key genes, from which 21 miRNAs and 55 TFs targeting key genes were predicted. In the miRNA network, numerous miRNAs targeting the ABR were involved in PCT onset and development regulation, for instance; miR-34-5p (as a plasma exosomal miRNA) may assist identify benign malignant thyroid nodules and is promising for early liquid biopsy of PTC (74). Another study found that miR-34a-5p induction promotes apoptosis and inhibits human PTC cell line proliferation and migration (K1 and TPC-1) (75). The n384546/miR-145-5p/AKT3 pathway (where miR-145-5p is located), may inhibit PTC cell proliferation, invasion and migration *in vitro* and *in vivo* (76). MiR-506-3p may inhibit PTC proliferation by suppressing YAP1 expression and regulating the YAP1-CDK2/Cy clinical E1 cell cycle pathway (77). MiR-449a may partially inhibit PTC progression by downregulating metamucin (MTDH) (78). Numerous miRNAs targeting ABR hold diagnostic and therapeutic significance for PTC, and the *ABR* gene, as one of the prognostic genes of PTC, has not been targeted in relevant studies and deserves further exploration.

In the transcription factor network, all three prognostic genes were regulated by *EGR1* (TF). Guo and Zhang (79) reported that the expression of EGR1/2 affects the proliferation of PTC cells and is related to poor prognosis(79). The transcription factor, *Yin Yang 1 (YY1)*, that targets ADH1B, was used to identify 88% patients with PTC and is expected to play a role in the differential diagnosis of PTC (80). Co-targeting *MYC* that regulates SERPINA1 and ABR can promote PTC proliferation by regulating ANXA1 and repressing lncRNA PAX8-AS1:28 expression (81, 82). Androgen receptor (AR) expression changes affect PTC progression. Furthermore, AR axis suppression in PTC patients may contribute to the aggressive behavior of PTC (83). Higher ESR1 expression in PTC is associated with poorer prognosis and lower overall survival in women (84). SMAD3-activation of the TGF-ß/Smad3 pathway, which jointly targets ABR and LPAR5, could alter tumor cell function in PTC by suppressing NIS expression and altering tumor cell function (85). Another related gene, *RUNX1*, was upregulated in PTC tissues and expression levels correlated with PTC staging. *RUNX1* knockdown could inhibit PTC proliferation, metastasis, and invasion (86).

Finally, we explored compounds that effectively target the four key genes. Currently, few studies within the DGIdb database report potential targeting compounds of these four key genes. Among compounds targeting ADH1B, alcohol intake was negatively associated with PTC cancer risk (87). Fomepizole inhibits the initial metabolism of ethylene glycol and methanol *via* ADH1B inhibition (88). There is no related research on acetaldehyde and abacavir that warrants further discussion. Meanwhile, human alpha-1-proteinase inhibitor (89), a potential targeting compound of SERPINA1, elicits a therapeutic effect on progressive ultimate fatal emphysema by inhibiting neutrophil elastase in the lung. Additionally, CHEMBL1630084, which blocked melanoma lung metastasis *via* LPA5 activation in a murine model (90). LPAR5 was highly expressed in PTC, and the lungs are important metastatic target organs related to PTC. Thus, CHEMBL1630084 and LPAR5 may provide protective effects against PTC pulmonary metastasis, and warrants further investigation. Furthermore, arachidonoylglycine has diagnostic potential for PTC (91), but the specific reason has not been studied. In this study, arachidonoylglycine was a compound targeting LPAR5, which provides insights for the study of related diagnostic mechanisms.

Although we performed a rigorous bioinformatics analysis, the number of PTC samples in the HT dataset was limited. To increase the credibility of the results, we verified the expression of key genes in clinical samples from patients with PTC in HT using qRT-PCR and IHC. However, there are few limitations in this study, including the small sample size. In the future, we will conduct a comparative analysis of PTC with HT vs HT by incorporating more HT samples, and expand our sample size to facilitate a more in-depth analysis of the genetic differences and similarities in patients with PTC and HT.

## 5 Conclusions

Using comprehensive bioinformatics methods, qRT-PCR, and IHC, we successfully screened, analyzed, and verified four key genes shared between HT and PTC. Specifically, *ABR, LPAR5*, and *SERPINA1* were significantly up-regulated, while *ADH1B* was significantly down-regulated in clinical PTC samples compared to normal samples. Furthermore, we investigated the underlying regulatory mechanism of these four important genes as well as prospective drugs targeting them. We evaluated the common pathogenesis and molecular mechanism underlying the occurrence of HT and PTC and provided a foundation for further characterizing the relationship in these conditions. In conclusion, our findings advance the current understanding of the molecular etiopathogenesis of HT and PTC, and support the potential clinical applications of candidate genes in their treatment.

## Data availability statement

The datasets presented in this study can be found in online repositories. The names of the repository/repositories and accession number(s) can be found in the article/**Supplementary Material**.

## Ethics statement

The studies involving human participants were reviewed and approved by the ethics committee of scientific research and clinical trial of The First Affiliated Hospital of Zhengzhou University. The patients/participants provided their written informed consent to participate in this study. Written informed consent was obtained from the individual(s) for the publication of any potentially identifiable images or data included in this article.

## Author contributions

TTL performed the data analysis and wrote the manuscript. NW, NL, and GD contributed to the data analysis and manuscript revision. MFP contributed to the literature search and data extraction. NW and DTY proofread the manuscript. TTL and DTY conceived and designed the study. All authors have contributed to the article and have approved the submitted version.

## Supplementary material

Supplementary Figure 1
**(A)** Receiver operating characteristic **(**ROC) curves of the four key genes (*ADH1B, ABR, SERPINA1*, and *LPAR5*) between papillary thyroid carcinoma (PTC) and normal samples in the GSE33630 dataset. **(B)** ROC curves of the four key genes between Hashimoto’s thyroiditis (HT) and normal samples in the GSE138198 dataset.

Supplementary Figure 2 The miRNA/TF-key gene-drug network created based on common key genes in papillary thyroid carcinoma (PTC) and Hashimoto’s thyroiditis (HT). The orange arrows represent the key genes in PTC and HT, the green diamonds represent miRNAs, the purple circles represent the transcription factors (TFs), and the red rectangles represent the compounds.
