# Supplementary figures and images for: Identifying and analyzing the key genes shared by papillary thyroid carcinoma and Hashimoto’s thyroiditis using bioinformatics methods
Source: Front Endocrinol (Lausanne). 2023 May 31;14:1140094. doi: 10.3389/fendo.2023.1140094 (PMC10266228; doi:10.3389/fendo.2023.1140094)

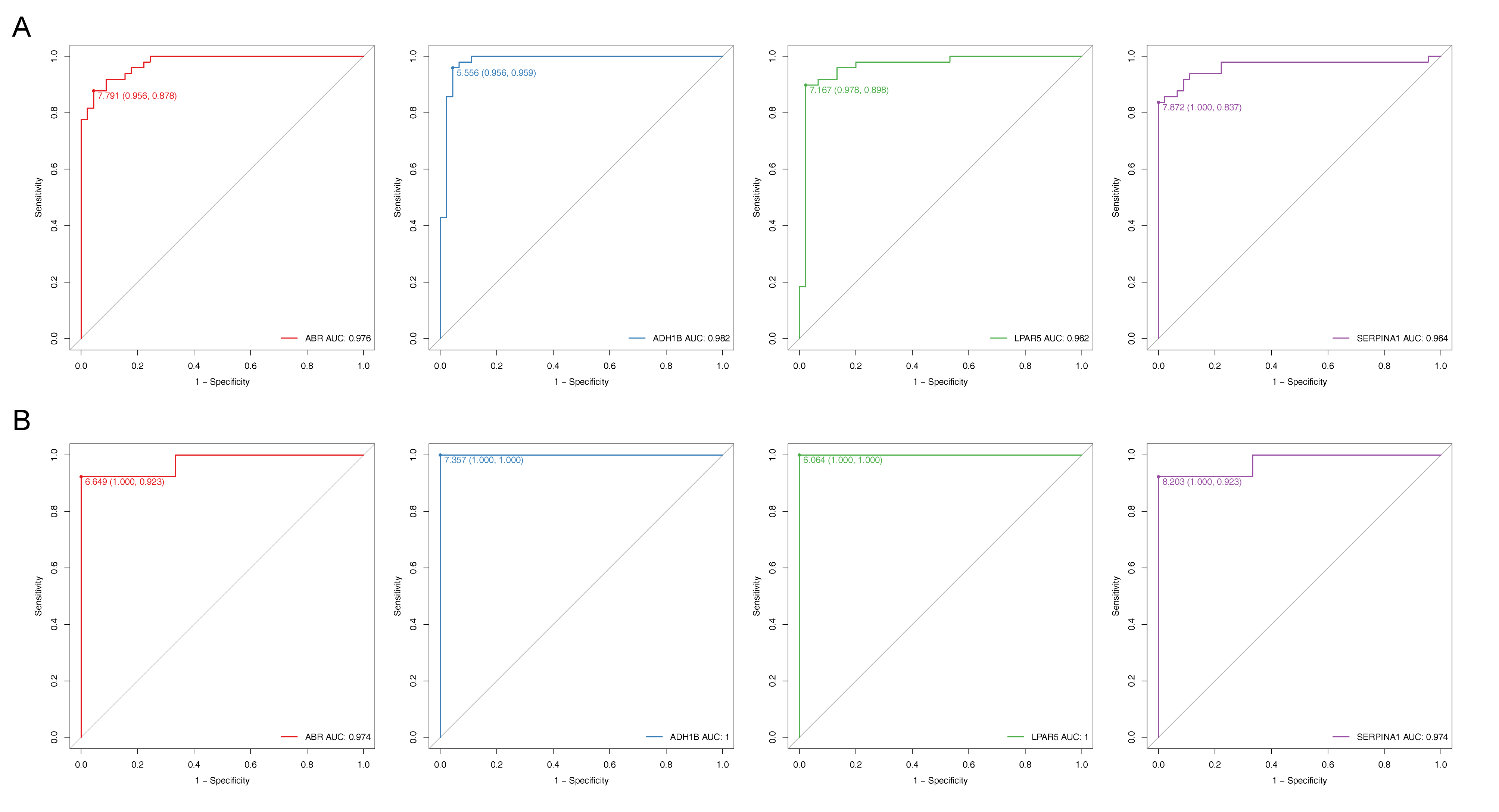

Supplement: Supplementary Figure 1 — (A) Receiver operating characteristic (ROC) curves of the four key genes (ADH1B, ABR, SERPINA1, and LPAR5) between papillary thyroid carcinoma (PTC) and normal samples in the GSE33630 dataset. (B) ROC curves of the four key genes between Hashimoto’s thyroiditis (HT) and normal samples in the GSE138198 dataset. [file Image_1.tif]

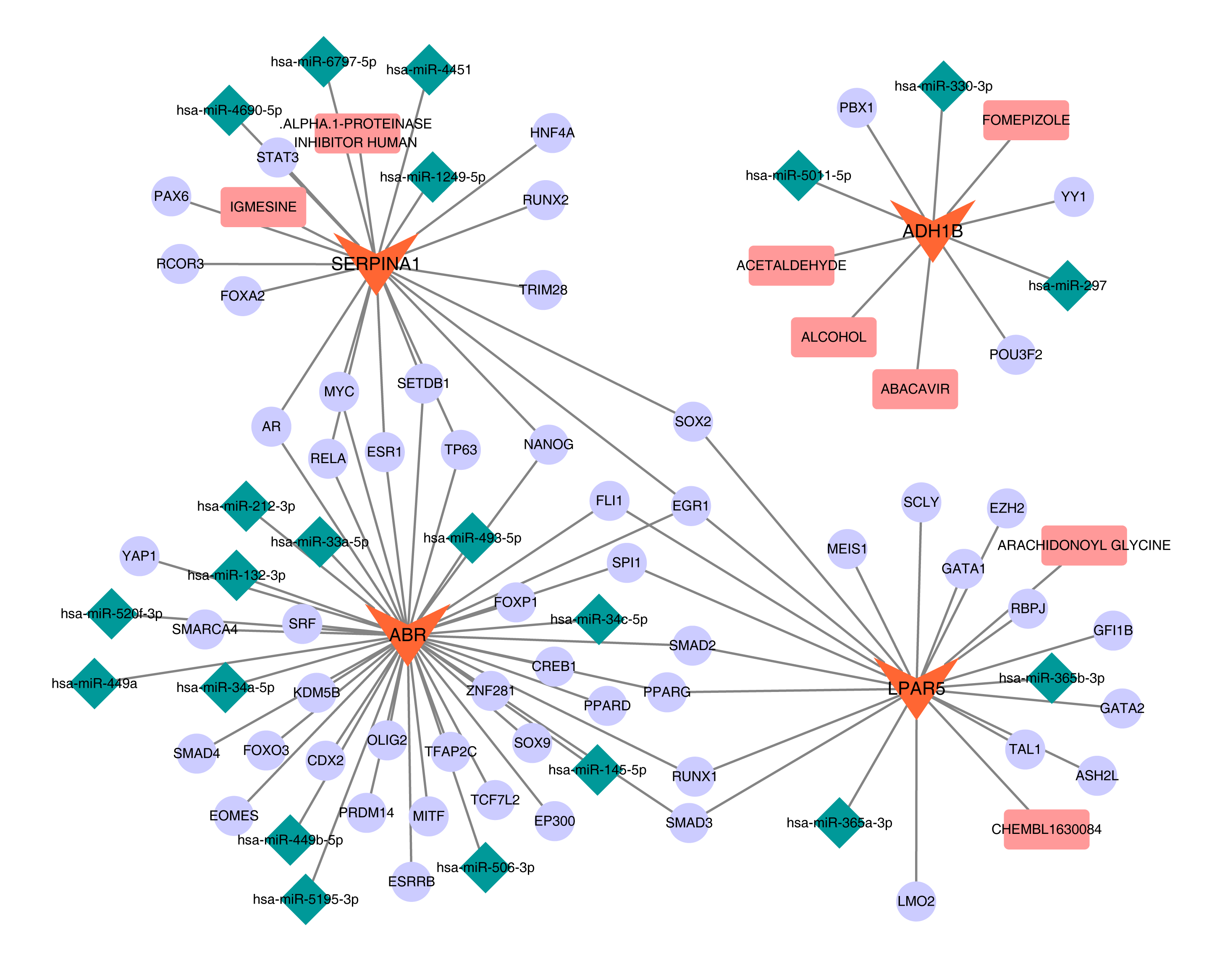

Supplement: Supplementary Figure 2 — The miRNA/TF-key gene-drug network created based on common key genes in papillary thyroid carcinoma (PTC) and Hashimoto’s thyroiditis (HT). The orange arrows represent the key genes in PTC and HT, the green diamonds represent miRNAs, the purple circles represent the transcription factors (TFs), and the red rectangles represent the compounds. [file Image_2.tif]
