# Supplementary material for: Identifying and analyzing the key genes shared by papillary thyroid carcinoma and Hashimoto’s thyroiditis using bioinformatics methods
Source: Front Endocrinol (Lausanne). 2023 May 31;14:1140094. doi: 10.3389/fendo.2023.1140094 (PMC10266228; doi:10.3389/fendo.2023.1140094)
Supplement: Supplementary file 3 [file Table_1.docx]

**Table 1 The primer sequences for RT-qPCR**

| **primer** | **sequence** |
| --- | --- |
| ABR For | GCCGTCTTCGATGCCAATAAC |
| ABR Rev | TGGGTAGAGTCGGTCCGTGAG |
| ADH1B For | CATCAACCCTCAAGACTACAAGAA |
| ADH1B Rev | GCGTCCAGTCAGTAGCAGCATAG |
| LPAR5 For | TGCTGTGCTTCGTGCCCTAC |
| LPAR5 Rev | GCGGACCTTTCGGATTGC |
| SERPINA1 For | CGTGAAGGTGCCTATGATGAAG |
| SERPINA1 Rev | CCAGTAATGGACAGTTTGGGTAA |
| GAPDH For | CCCATCACCATCTTCCAGG |
| GAPDH Rev | CATCACGCCACAGTTTCCC |
